# Effects of type 2 diabetes mellitus on the pharmacokinetics of berberine in rats

**DOI:** 10.1080/13880209.2016.1255649

**Published:** 2016-12-09

**Authors:** Yuzhen Jia, Binger Xu, Jisen Xu

**Affiliations:** Department of Pediatrics, Yidu Central Hospital of Weifang, Shandong, China

**Keywords:** Berberine, LC-MS/MS, Pharmacokinetics, T2DM

## Abstract

**Context:** Berberine is an active alkaloid isolated from *Rhizoma coptidis* [*Coptis chinensis* Franch. (Ranunculaceae)] that is widely used for the treatment of diabetes, hyperlipidemia and hypertension. However, the pharmacokinetics of berberine in normal rats and type 2 diabetes mellitus (T2DM) model rats are not clear.

**Objective:** This study compares the pharmacokinetics of berberine between normal and T2DM model rats.

**Materials and methods:** The T2DM model rats were fed with high fat diet for 4 weeks, induced by low-dose (30 mg/kg) streptozotocin for 72 h and validated by determining the peripheral blood glucose level. Rats were orally treated with berberine at a dose of 20 mg/kg and then berberine concentration in rat plasma was determined by employing a sensitive and rapid LC-MS/MS method.

**Results:** The significantly different pharmacokinetic behaviour of berberine was observed between normal and T2DM model rats. When compared with the normal group, *C*_max_, *t*_1/2_ and AUC_(0–_*_t_*_)_ of berberine were significantly increased in the model group (17.35 ± 3.24 vs 34.41 ± 4.25 μg/L; 3.95 ± 1.27 vs 9.29 ± 2.75 h; 151.21 ± 23.96 vs 283.81 ± 53.92 μg/h/L, respectively). In addition, oral clearance of berberine was significantly decreased in the model group (134.73 ± 32.15 vs 62.55 ± 16.34 L/h/kg).

**Discussion and conclusion:** In T2DM model rats, the pharmacokinetic behaviour of berberine was significantly altered, which indicated that berberine dosage should be modified in T2DM patients.

## Introduction

Diabetes mellitus is one kind of metabolic syndromes characterized by chronic hyperglycemia (Atkinson & Maclaren 1994; Ismail-Beigi 2012). Diabetes cases fall into two broad pathogenetic categories: type I and type II diabetes mellitus (T1DM and T2DM) (Tan & Cheah 1990; Hu 2011). T2DM, which is previously referred to as noninsulin-dependent diabetes mellitus, accounts for approximately 90% of the diabetes patients (Al-Amer et al. 2015; Tangvarasittichai 2015). One of the significant features of diabetes mellitus is lipid metabolism malfunction, which results in hyperlipidemia (Moest et al. 2013). In the current clinical treatment, western medicines are extensively used to control hyperglycaemia, hyperlipidaemia and insulin resistance of type 2 diabetes mellitus, such as sulfonylurea, biguanides, thiazolidinediones and glycosidase inhibitors (Shankar et al. 2015). However, the clinical efficacy of these drugs in T2DM treatment is limited and these drugs are very expensive.

Berberine is a pharmacological component isolated form *Rhizoma coptidis* [*Coptis chinensis* Franch. (Ranunculaceae)], which possesses a variety of activities, such as antitumour, anti-inflammatory, anti-atherosclerosis and treating infectious diarrhoea (Lao-ong et al. 2012; Liu et al. 2015; Orvos et al. 2015; Park et al. 2015; Li et al. 2016). Recent studies show that berberine can be used for controlling arrhythmia, lowering blood lipid, lowering blood pressure and reducing blood sugar in clinical studies (Lei et al. 2011; Ortiz et al. 2014; Chang et al. 2015). Furthermore, it can effectively promote the islet cells regeneration and contribute to islet function recovery (Liu et al. 2014). Therefore, berberine is widely used for diabetes, hyperlipidaemia and hypertension treatment (Wu et al. 2014). Animal experiments have proved that berberine is able to inhibit hepatic gluconeogenesis, so as to improve fasting blood sugar levels in diabetic mice without dependence on insulin levels (Zhang et al. 2012; Ren et al. 2013; Jiang et al. 2015).

Only the pharmacokinetics of berberine has been reported among healthy animals, while little attention is paid to the pharmacokinetics of berberine in T2DM animals (Chen et al. 2015; Li et al. 2015; Liu et al. 2015). It is well-known that patients are ultimate consumers of drugs. Therefore, it is necessary and important to study the pharmacokinetics of drugs in the pathological state. In the present study, T2DM model rats were induced by high fat diet and low-dose streptozotocin (STZ). In the next step, the pharmacokinetics of berberine was compared in normal rats and T2DM model rats by adopting a sensitive and reliable LC-MS/MS method.

## Materials and methods

### Chemicals and reagents

Berberine hydrochloride (purity >97%) and hydrocortisone were obtained from the national institute for the control of pharmaceutical and biological products (Beijing, China). Acetonitrile was purchased from Fisher Scientific (Fair Lawn, NJ). Streptozotocin was purchased from Sigma Chemical Co. (St. Louis, MO). Formic acid was purchased from Anaqua Chemicals Supply Inc. Limited (Houston, TX). Ultrapure water was prepared with a Milli-Q water purification system (Millipore, Billerica, MA). All other chemicals were of analytical grade or better.

### Animals and diets

Male Sprague-Dawley (SD) rats weighing 220–250 g were provided by the experimental animal centre of the Weifang Medical College (Weifang, China). Rats were bred in a breeding room at 25 °C, 60 ± 5% humidity and a 12 h dark–light cycle. Tap water and normal chow were given *ad libitum*. All the experimental animals were housed under the above conditions for a 5-day acclimation, and were fasted overnight before the experiments.

The conventional diet and the high fat diet (HFD) were provided by the laboratory animal centre of Weifang Medical College. The conventional diet contains 41.47% carbohydrate, 14.42% fat and 21.06% protein. The HFD contains 10% lard, 20% sucrose, 1% choline chloride, 2.5% cholesterol and 66.5% conventional diet.

### Establishment of T2DM model rats

After adaptive feeding for one week, the rats were divided into two groups of six rats in each group as follows: group 1, normal control rats; group 2, T2DM model rats. T2DM model rats were established as previously reported (Sun et al. 2016; Zhai et al. 2016). The rats of normal control group were fed with conventional diet. Rats of group 2 were fed with HFD. After four weeks, the rats of group 2 were injected intraperitoneally with low dose (30 mg/kg) of STZ which was prepared in sterile citrate buffer (w/v: 2%). Six rats of the normal control group were injected intraperitoneally with the same volume of sterile citrate buffer. After 72 h, the peripheral blood glucose level in tails of rats was detected by the Precision Xtra blood glucose monitoring system (Alameda, CA). The model was successfully established if the blood glucose of the rats was over 16.7 mmol/L and polyuria and polydipsia appeared in these rats. After the levels of blood glucose stably were maintained for one week, the blood glucose and serum insulin concentrations of the rats in each group were determined.

### Instrumentation and conditions

The analysis was performed on an Agilent 1290 series liquid chromatography system (Agilent Technologies, Palo Alto, CA), and an Agilent 6460 triple-quadruple mass spectrometer (Agilent Technologies, CA) with Turbo Ion spray. The chromatographic separation of berberine was performed on Waters X-Bridge C18 column (3.0 × 100 mm, i.d.; 3.5 μm) at room temperature. The mobile phase was water (containing 0.1% formic acid) and acetonitrile (40:60, v: v) at a flow rate of 0.4 mL/min.

The mass scan mode was positive MRM mode. The precursor ion and product ion are *m/z* 336.2 → 320.2 for berberine and *m/z* 363.5 → 121.0 for IS, respectively. The collision energy for berberine and IS were 30 and 25 ev, respectively. The MS/MS conditions were optimized as follows: fragmentor, 140 V; capillary voltage, 4 kV; Nozzle voltage, 500 V; nebulizer gas pressure (N_2_), 40 psig; drying gas flow (N_2_), 10 L/min; gas temperature, 350 °C; sheath gas temperature, 400 °C; sheath gas flow, 11 L/min.

### Pharmacokinetic study

The animal facilities and protocols were approved by the Institutional Animal Care and Use Committee. All procedures were in accordance with the national institute of health guidelines regarding the principles of animal care. After the levels of blood glucose stably were maintained for one week, then the rats were fasted for 12 h with free access to water prior to the pharmacokinetic study. Berberine was dissolved in oral suspension and orally administered to rats at a dose of 20 mg/kg. Blood samples (200 μL) were collected into a heparinized tube via the oculi chorioideae vein at 0.083, 0.167, 0.33, 0.5, 1, 2, 4, 6, 8, 12 and 24 h, respectively. After centrifugation at 4000 rpm for 5 min, plasma samples were obtained and frozen at −40 °C until analysis.

### Plasma sample preparation

The plasma (100 μL) was spiked with 10 μL of IS (100 ng/mL), and then the mixture was extracted with 190 μL of acetonitrile by vortex for 1 min. After centrifugation (15,000 rpm) for 10 min, the supernatant was injected into the LC-MS/MS system for quantitative analysis.

### Preparation of standard and quality control samples

A stock solution of berberine was prepared in acetonitrile at a concentration of 2 mg/mL. The stock solution of IS was prepared in acetonitrile at a concentration of 100 ng/mL. Calibration standard samples for berberine were prepared in blank rat plasma at concentrations of 1, 2, 5, 10, 20, 50 and 100 ng/mL. The quality control (QC) samples were prepared at low (2 ng/mL), medium (10 ng/mL) and high (75 ng/mL) concentrations in the same way as the plasma samples for calibration, and QC samples were stored at −40 °C until analysis.

### Method validation

The method validation assay was performed according to the United States Food and Drug Administration (FDA) guidelines. Selectivity was investigated by comparing the chromatograms of six different batches of blank rat plasma with the corresponding spiked plasma to monitor interference of endogenous substances and metabolites. To obtain the calibration curve, seven concentrations of the calibration standard were processed and determined as described above. The linearity of calibration curves was constructed by plotting peak area ratios (*y*) of the analytes to IS against the nominal concentration (*x*) of analytes with weighted (1/*x*^2^) least square linear regression. The lower limit of detection (LLOD) and lower limit of quantification (LLOQ) were determined as the concentration of the analyte with a signal-to-noise ratio at 3 and 10, respectively. The intra-day precision and accuracy of the method were confirmed by determining QC samples at three different concentrations five times on a single day, and the inter-day precision and accuracy were assessed by determining the QC samples over three consecutive days. For each concentration, five replicates were prepared. Relative standard deviation (RSD) and relative error (RE) were used to express the precision and accuracy, respectively.

The extraction recovery was assessed by comparing peak areas obtained from extracted spiked samples with those originally spiked in the blank plasma samples. The matrix effect was evaluated by comparing the peak areas of the post-extracted spiked QC samples with those of corresponding standard solutions. These procedures were repeated for five replicates at three QC concentration levels. For sample stability, three levels of QC samples were determined under different conditions, including short-term stability at room temperature for 24 h, long-term stability at −40 °C for 30 days, and three freeze-thaw cycles at −40 °C.

### Pharmacokinetic data analysis

The pharmacokinetic parameters, including area under the plasma concentration–time curve (*AUC*), maximal plasma concentration (*C*_max_), the time for maximal plasma concentration (*T*_max_) and mean residence time (*MRT*) were calculated using DAS 3.0 pharmacokinetic software (Chinese Pharmacological Association, Anhui, China).

### Data analysis

The statistical difference was calculated using an unpaired *t*-test with a two-tailed distribution for comparison of two mean values. A *p* value <0.05 was considered statistically significant.

## Results and discussion

### Establishment of T2DM model rats

After STZ injection for 72 h, the average blood glucose concentration of rats in T2DM model group reached 16.95 mmoL/L. Subsequently, blood glucose levels were stably maintained for one week. The blood glucose and serum insulin concentrations of the rats are shown in Table 1. In T2DM model group, the blood glucose concentration of rats was significantly higher, while serum insulin concentration was much lower when compared with the control group, which indicated that the rats suffered from T2DM induced by STZ. In addition, the apathetic state and behaviours of polyuria and polydipsia appeared in T2DM model group. Therefore, T2DM model rats were successfully established.

**Table 1.** The blood glucose and serum insulin concentrations of the rats after the blood glucose levels were stably maintained for one week.

Group	Normal group	T2DM model group
Blood glucose (mmol/L)	4.38 ± 0.97	16.95 ± 2.42*
Serum insulin (mIU/L)	16.54 ± 1.15	12.21 ± 1.87*

* *p* < 0.05, compared with normal group.

### Sample preparation

Due to the heterogeneous nature of plasma, a sample pretreatment procedure is often needed to remove protein and potential interferences before LC-MS/MS analysis. Currently, the most widely used biological sample preparation methods are protein precipitation, solid phase extraction and liquid–liquid extraction. All the sample pretreatment methods were investigated to achieve good resolution and high recovery of analyte from spiked biologic matrices. Finally, protein precipitation was selected for berberine. The highest recovery was obtained using acetonitrile as a protein precipitant.

### Chromatography and mass spectrometry

**Figure 1.**
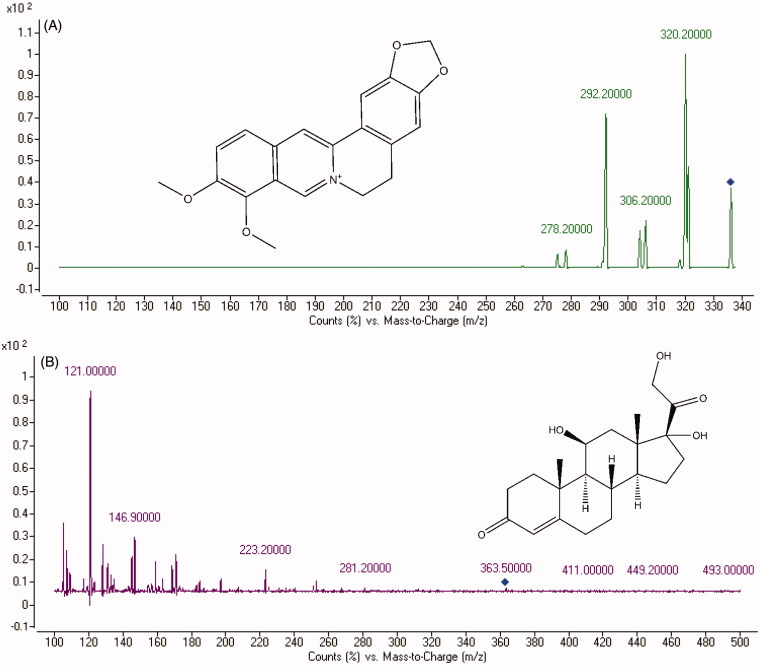
The mass spectra of (a) berberine (b) and IS.

**Figure 2.**
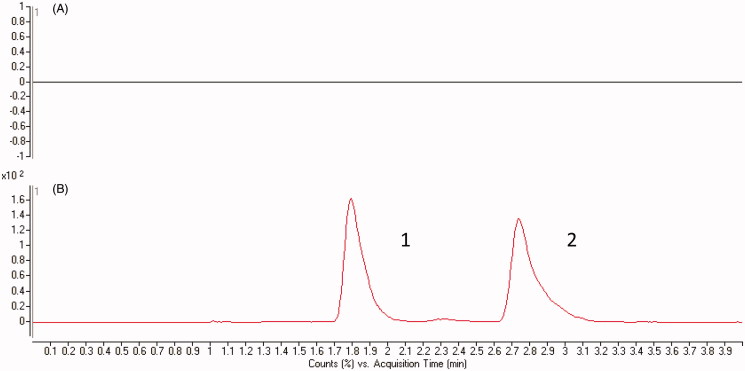
Chromatograms of (a) blank plasma, and (b) plasma spiked with berberine and IS. B1: Berberine; B2: IS.

A reliable and sensitive method to determine the concentration of berberine had been established in this research. This newly established method enabled us to determine the concentration of berberine *in vivo* after oral administration of berberine. As shown in Figure 1, the optimized mass transition ion-pairs for quantification, including precursor and product ions, were *m/z* 336.2 → 320.2 for berberine and *m/z* 363.5 → 121.0 for IS, respectively. Blank plasma, plasma spiked with berberine and hydrocortisone are shown in Figure 2. No significant interference substances were observed at the retention time of berberine in plasma samples.

### Method validation

The standard curve for berberine in plasma was linear in the concentration range of 1–100 ng/mL with correlation coefficient values >0.998. The LLOQ and LLOD were 1 and 0.35 ng/mL, respectively.

Intra-day and inter-day precision and accuracy were determined by measuring six replicates of QC samples at three concentration levels in rat plasma. The intra-day and inter-day precision and accuracy data are shown in Table 2. These results demonstrated that the precision and accuracy values were well within an acceptable range of 15%.

**Table 2.** The intra-day and inter-day precision and accuracy of berberine in plasma samples.

		Intra-day			Inter-day		
Analyte	Plasma samples (ng/ml)	Concentrationmeasured (ng/ml)	Precision(%, RSD)	Accuracy(%, RE)	Concentrationmeasured (ng/ml)	Precision(%, RSD)	Accuracy(%, RE)
Berberine	2	1.85	4.58	−7.50	2.17	5.64	8.50
	10	10.64	3.24	6.40	9.67	7.56	6.70
	50	46.68	6.55	−6.64	53.64	8.27	7.28

The mean extraction recoveries determined using three replicates of QC samples at three concentration levels in rat plasma were 87.6 ± 4.9, 92.3 ± 5.4 and 89.6 ± 6.5% for 2, 10 and 50 ng/mL, respectively.

For ionization, the peak areas of berberine after spiking evaporated plasma samples at three concentration levels were comparable to those of similarly prepared aqueous standard solutions (ranging from 92.5 to 106.7%), suggesting that there was no measurable matrix effect that interfered with berberine determination in rat plasma.

The stability of berberine in plasma was evaluated by analyzing three replicates of quality control samples containing 2, 10 and 75 ng/mL berberine after short-term storage (25 °C, 24 h), long-term cold storage (−40 °C, 30 days) and within three freeze (−40 °C)-thaw (room temperature) cycles. As shown in Table 3, all the samples displayed 90–110% recoveries after various stability tests. Taken together, the above results show that a sensitive and reliable method for analyzing berberine in rat plasma has been developed and validated and can be used to investigate the pharmacokinetics of berberine in normal and T2DM model rats.

**Table 3.** Stability of berberine in plasma samples (*n* = 3).

		Stability (%, RE)		
Analyte	Plasma samples(ng/ml)	Short-term (24 h atroom temperature)	Long-term(30 days at −40 °C)	Three freeze (−40 °C)–thaw(room temperature) cycles
Berberine	2	6.42	−5.22	7.62
	10	8.94	6.84	−5.84
	50	8.56	−9.35	7.55

### Pharmacokinetic study

**Figure 3.**
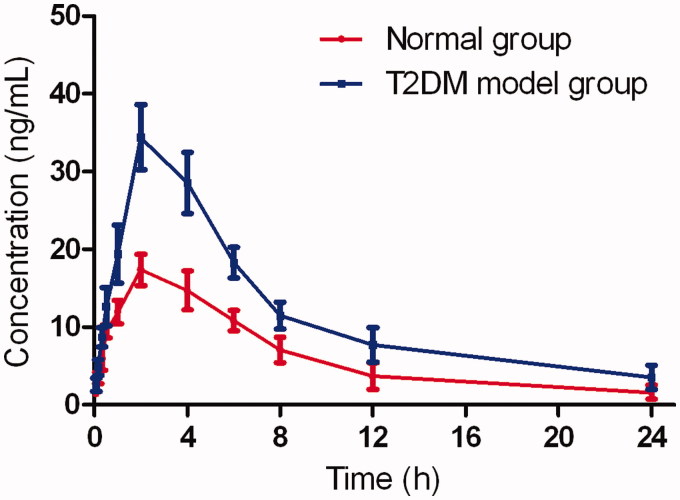
The pharmacokinetic profiles of berberine in rats after oral administration of berberine in normal group and T2DM model group.

The validated analytical method was employed to study the pharmacokinetic behaviours of berberine in normal and T2DM model rats. The mean plasma concentration–time curves of berberine after oral administration of berberine are shown in Figure 3.

The pharmacokinetic parameters were calculated using the non-compartmental method with DAS 3.0 pharmacokinetic software (Chinese Pharmacological Association, Anhui, China). The pharmacokinetic parameters are shown in Table 4.

**Table 4.** Pharmacokinetic parameters of berberine in rats after oral administration of berberine to normal rats and T2DM model rats (20 mg/kg; *n* = 6, Mean ± S.D.).

Parameter	Normal group	T2DM group
*T*_max_*(h)*	2.04 ± 0.16	2.24 ± 0.21
*C*_max_*(μg/L)*	17.35 ± 3.24	34.41 ± 4.25
**t*_*1/2*_*(h)**	3.95 ± 1.27	9.29 ± 2.75
AUC *(*0*–t) (μg/h/L)*	151.21 ± 23.96	283.81 ± 53.92
AUMC *(*0*–t) (μg/h/L)*	1067.21 ± 224.84	2097.21 ± 543.57
CL *(L/h/kg)*	134.73 ± 32.15	62.55 ± 16.34
MRT *(h)*	6.94 ± 1.15	7.32 ± 1.47

The results showed that both in the normal control and T2DM model group, berberine was absorbed rapidly into the body at 30 min after oral administration of berberine hydrochloride. Moreover, it was noteworthy that in comparison to the normal group, the peak concentration, terminal half-life and the area under the plasma drug concentration–time curves of berberine were significantly increased in T2DM model group (17.35 ± 3.24 vs 34.41 ± 4.25 μg/L; 3.95 ± 1.27 vs 9.29 ± 2.75 h; 151.21 ± 23.96 vs 283.81 ± 53.92 μg/h/L, respectively) in comparison to the normal group. When compared with the normal group (134.73 ± 32.15 L/h/kg), the remarkable decrease of CL/F of berberine in T2DM model group (62.55 ± 16.34 L/h/kg) suggested that the elimination of berberine slowed down.

Insulin resistance, hyperglycaemia, impaired glucose tolerance, hyperlipidaemia, hypertension and even blood circulation decrease often occur in T2DM rats, which leads to the change of haemorrheological parameters (Aypak et al. 2015; Franch-Nadal et al. 2015; Oh et al. 2016). As berberine is mainly absorbed in intestine and then distributed to different organs (Ma et al. 2011), the change of haemorrheological parameters will affect the absorption in intestine and the distribution in different organs. Therefore, pharmacokinetic parameters of berberine will change in T2DM model rats. Many research articles have also demonstrated that the disease condition will influence the pharmacokinetic parameters of the compounds (Chen et al. 2013; Liu et al. 2014; Gong et al. 2015). Thus, it suggested that berberine dosage should be modified in T2DM patients.

## Conclusions

In this study, the pharmacokinetics of berberine in normal and T2DM model rats were investigated using a sensitive and reliable LC-MS/MS method. There were statistically significant differences in pharmacokinetic parameters of berberine including the *C*_max_, *t*_1/2_, AUC_(0–_*_t_*_)_, AUC_0–_*_t_* and CL/*F* between the normal and T2DM model rats after oral administration with berberine hydrochloride. The results indicated that the rate and extent of drug metabolism was altered in rats with T2DM, which suggested us that the dosage of berberine should be modified in T2DM patients.

## References

[CIT0001] Al-Amer R, Ramjan L, Glew P, Salamonson Y. 2015 Diagnosis of type 2 diabetes: the experience of Jordanian patients with co-existing depression. Issues Ment Health Nurs. 36:231–238. 25897571 10.3109/01612840.2014.960627

[CIT0002] Atkinson MA, Maclaren NK. 1994 The pathogenesis of insulin-dependent diabetes mellitus. N Engl J Med. 331:1428–1436. 7969282 10.1056/NEJM199411243312107

[CIT0003] Aypak C, Turedi O, Bircan MA, Araz M. 2015 Association of haematological parameters with bone mineral density in elderly diabetic women. Acta Clin Belg. 70:339–344. 25982378 10.1179/2295333715Y.0000000030

[CIT0004] Chang W, Chen L, Hatch GM. 2015 Berberine as a therapy for type 2 diabetes and its complications: From mechanism of action to clinical studies. Biochem Cell Biol. 93:479–486. 25607236 10.1139/bcb-2014-0107

[CIT0005] Chen G, Lu F, Xu L, Dong H, Yi P, Wang F, Huang Z, Zou X. 2013 The anti-diabetic effects and pharmacokinetic profiles of berberine in mice treated with Jiao-Tai-Wan and its compatibility. Phytomedicine. 20:780–786. 23582408 10.1016/j.phymed.2013.03.004

[CIT0006] Chen Y, Li Y, Wang Y, Yang Q, Dong Y, Weng X, Zhu X, Gong Z, Zhang R. 2015 Comparative pharmacokinetics of active alkaloids after oral administration of Rhizoma Coptidis extract and Wuji Wan formulas in rat using a UPLC-MS/MS method. Eur J Drug Metab Pharmacokinet. 40:67–74. 24577954 10.1007/s13318-014-0181-1

[CIT0007] Franch-Nadal J, Roura-Olmeda P, Benito-Badorrey B, Rodriguez-Poncelas A, Coll-de-Tuero G, Mata-Cases M. 2015 Metabolic control and cardiovascular risk factors in type 2 diabetes mellitus patients according to diabetes duration. Fam Pract. 32:27–34. 25194144 10.1093/fampra/cmu048

[CIT0008] Gong Z, Chen Y, Zhang R, Yang Q, Wang Y, Guo Y, Zhou B, Weng X, Liu X, Li Y, et al 2015 Pharmacokinetic difference of berberine between normal and chronic visceral hypersensitivity irritable bowel syndrome rats and its mechanism. Arch Pharm Res. 38:1888–1896. 25716428 10.1007/s12272-015-0568-9

[CIT0009] Hu FB. 2011 Globalization of diabetes: the role of diet, lifestyle, and genes. Diabetes Care. 34:1249–1257. 21617109 10.2337/dc11-0442 PMC3114340

[CIT0010] Ismail-Beigi F. 2012 Clinical practice. Glycemic management of type 2 diabetes mellitus. N Engl J Med. 366:1319–1327. 22475595 10.1056/NEJMcp1013127

[CIT0011] Jiang SJ, Dong H, Li JB, Xu LJ, Zou X, Wang KF, Lu FE, Yi P. 2015 Berberine inhibits hepatic gluconeogenesis via the LKB1-AMPK-TORC2 signaling pathway in streptozotocin-induced diabetic rats. World J Gastroenterol. 21:7777–7785. 26167077 10.3748/wjg.v21.i25.7777 PMC4491964

[CIT0012] Lao-ong T, Chatuphonprasert W, Nemoto N, Jarukamjorn K. 2012 Alteration of hepatic glutathione peroxidase and superoxide dismutase expression in streptozotocin-induced diabetic mice by berberine. Pharm Biol. 50:1007–1012. 22775417 10.3109/13880209.2012.655377

[CIT0013] Lei G, Dan H, Jinhua L, Wei Y, Song G, Li W. 2011 Berberine and itraconazole are not synergistic *in vitro* against *Aspergillus fumigatus* isolated from clinical patients. Molecules. 16:9218–9233. 22051933 10.3390/molecules16119218 PMC6264531

[CIT0014] Li G, Yang F, Liu M, Su X, Zhao M, Zhao L. 2015 Development and application of a UPLC-MS/MS method for simultaneous determination of fenofibric acid and berberine in rat plasma: application to the drug-drug pharmacokinetic interaction study of fenofibrate combined with berberine after oral administration in rats. Biomed Chromatogr. 30:1075–1082. 10.1002/bmc.3652 26577601

[CIT0015] Li H, Liu L, Xie L, Gan D, Jiang X. 2016 Effects of berberine on the pharmacokinetics of losartan and its metabolite EXP3174 in rats and its mechanism. Pharm Biol. 54:2886–2894. 27327872 10.1080/13880209.2016.1190762

[CIT0016] Liu CH, Tang WC, Sia P, Huang CC, Yang PM, Wu MH, Lai IL, Lee KH. 2015 Berberine inhibits the metastatic ability of prostate cancer cells by suppressing epithelial-to-mesenchymal transition (EMT)-associated genes with predictive and prognostic relevance. Int J Med Sci. 12:63–71. 25552920 10.7150/ijms.9982 PMC4278877

[CIT0017] Liu L, Liu J, Gao Y, Yu X, Xu G, Huang Y. 2014 Uncoupling protein-2 mediates the protective action of berberine against oxidative stress in rat insulinoma INS-1E cells and in diabetic mouse islets. Br J Pharmacol. 171:3246–3254. 24588674 10.1111/bph.12666 PMC4080978

[CIT0018] Liu M, Su X, Li G, Zhao G, Zhao L. 2015 Validated UPLC-MS/MS method for simultaneous determination of simvastatin, simvastatin hydroxy acid and berberine in rat plasma: application to the drug-drug pharmacokinetic interaction study of simvastatin combined with berberine after oral administration in rats. J Chromatogr B Analyt Technol Biomed Life Sci. 1006:8–15. 10.1016/j.jchromb.2015.09.033 26519618

[CIT0019] Liu QF, Shi XJ, Li ZD, Zhong MK, Jiao Z, Wang B. 2014 Pharmacokinetic comparisons of berberine and palmatine in normal and metabolic syndrome rats. J Ethnopharmacol. 151:287–291. 24269776 10.1016/j.jep.2013.10.031

[CIT0020] Ma BL, Ma YM, Gao CL, Wu JS, Qiu FR, Wang CH, Wang XH. 2011 Lipopolysaccharide increased the acute toxicity of the *Rhizoma coptidis* extract in mice by increasing the systemic exposure to *Rhizoma coptidis* alkaloids. J Ethnopharmacol. 138:169–174. 21924335 10.1016/j.jep.2011.08.074

[CIT0021] Moest H, Frei AP, Bhattacharya I, Geiger M, Wollscheid B, Wolfrum C. 2013 Malfunctioning of adipocytes in obesity is linked to quantitative surfaceome changes. Biochim Biophys Acta. 1831:1208–1216. 24046861 10.1016/j.bbalip.2013.04.001

[CIT0022] Oh W, Kim E, Castro MR, Caraballo PJ, Kumar V, Steinbach MS, Simon GJ. 2016 Type 2 diabetes mellitus trajectories and associated risks. Big Data. 4:25–30. 27158565 10.1089/big.2015.0029 PMC4851215

[CIT0023] Ortiz LM, Lombardi P, Tillhon M, Scovassi AI. 2014 Berberine, an epiphany against cancer. Molecules. 19:12349–12367. 25153862 10.3390/molecules190812349 PMC6271598

[CIT0024] Orvos P, Virag L, Talosi L, Hajdu Z, Csupor D, Jedlinszki N, Szel T, Varro A, Hohmann J. 2015 Effects of *Chelidonium majus* extracts and major alkaloids on hERG potassium channels and on dog cardiac action potential: a safety approach. Fitoterapia. 100:156–165. 25481375 10.1016/j.fitote.2014.11.023

[CIT0025] Park SH, Sung JH, Kim EJ, Chung N. 2015 Berberine induces apoptosis via ROS generation in PANC-1 and MIA-PaCa2 pancreatic cell lines. Braz J Med Biol Res. 48:111–119. 25517919 10.1590/1414-431X20144293 PMC4321216

[CIT0026] Ren LM, Zhuo YJ, Hao ZS, He HM, Lu HG, Zhao D. 2013 Berberine improves neurogenic contractile response of bladder detrusor muscle in streptozotocin-induced diabetic rats. J Ethnopharmacol. 150:1128–1136. 24184080 10.1016/j.jep.2013.10.039

[CIT0027] Shankar RR, Xu L, Golm GT, O'Neill EA, Goldstein BJ, Kaufman KD, Engel SS. 2015 A comparison of glycaemic effects of sitagliptin and sulfonylureas in elderly patients with type 2 diabetes mellitus. Int J Clin Pract. 69:626–631. 25652751 10.1111/ijcp.12607

[CIT0028] Sun Z, Zhan L, Liang L, Sui H, Zheng L, Sun X, Xie W. 2016 ZiBu PiYin recipe prevents diabetes-associated cognitive decline in rats: possible involvement of ameliorating mitochondrial dysfunction, insulin resistance pathway and histopathological changes. BMC Complement Altern Med. 16:200. 27393392 10.1186/s12906-016-1177-y PMC4938951

[CIT0029] Tan KT, Cheah JS. 1990 Pathogenesis of type 1 and type 2 diabetes mellitus. Ann Acad Med Singapore. 19:506–511. 2221810

[CIT0030] Tangvarasittichai S. 2015 Oxidative stress, insulin resistance, dyslipidemia and type 2 diabetes mellitus. World J Diabetes. 6:456–480. 25897356 10.4239/wjd.v6.i3.456 PMC4398902

[CIT0031] Wu JS, Liu Y, Shi R, Lu X, Ma YM, Cheng NN. 2014 Effects of combinations of Xiexin decoction constituents on diabetic nephropathy in rats. J Ethnopharmacol. 157:126–133. 25278183 10.1016/j.jep.2014.09.024

[CIT0032] Zhai L, Gu J, Yang D, Hu W, Wang W, Ye S. 2016 Metformin ameliorates podocyte damage by restoring renal tissue nephrin expression in type 2 diabetic rats. J Diabetes. [Epub ahead of print]. doi: 10.1111/1753-0407.12437. 27248136

[CIT0033] Zhang M, Lv X, Li J, Meng Z, Wang Q, Chang W, Li W, Chen L, Liu Y. 2012 Sodium caprate augments the hypoglycemic effect of berberine via AMPK in inhibiting hepatic gluconeogenesis. Mol Cell Endocrinol. 363:122–130. 22922125 10.1016/j.mce.2012.08.006 PMC3795615

